# Hospital Finance

**Published:** 1922-08

**Authors:** 


### HOSPITAL FINANCE.

### CHARING CROSS HOSPITAL.

SPEAKING at the Annual General Court of Governors of Charing Cross Hospital, the Chairman, Mr. George Verity, said that a number of improvements to buildings and equipment had been effected during the year, *viz.*, the lift in the Nurses' Home; the renovation of the mortuary; the completion and re-equipment of the X-ray Department; the reconstruction of the electrical works; sundry works in offices, kitchens, etc.; and the repainting of the whole of the exterior ironwork. The expenditure on these works, approximately £5,100, had been financially digested in the year. A covered verandah had been erected for the Children's Ward, as a playground and a fresh-air cure, at the cost of a special benefactor. Extensions and alterations to kitchen and basement, and the painting and repairing of the whole of the woodwork of the façade, at an estimated cost of £1,180, were still in hand and would also be digested. During the last month the boilers had all broken down, and after taking the best advice and getting estimates, they had found it necessary to proceed with the work at a cost of approximately £500. They were then faced with serious structural defects in the building in two of the operating theatres, necessitating alterations estimated at some £800. They had applied to King Edward's Fund for a special donation, which they trusted would be granted, otherwise, at the end of the year, they would find their finances in jeopardy. These two "bombshells," which annulled their economies, were really heartbreaking. They were making great efforts to reduce expenditure, and hoped to have something to show next year. The cost per in-patient bed was nearly the lowest of any British hospital. A great extension to the Nurses' Home, a Maternity Ward and a new Massage Department, were still urgently needed. Unfortunately, the scheme of saving cost of rent and purchase of land, by building on top of the existing structure, had been condemned on grounds of safety by the consulting architects and the district surveyor. One of the events of the year had been the dedication of a ward to the Shanghai Race Club, the members of which, Englishmen some 10,000 miles away, had given £12,000 for endowment purposes, which meant £600 a year in dividends, and £500 to help with the running expenses. Since the end of the year under review a canteen had been installed in the out-patients' department by the Ladies' Guild, and was doing valuable work.

The total income of the hospital was £58,776, of which £12,234 represented patients' contributions. They had hoped for more, but in these times of stress and penury it was wonderful what their Lady Almoner, Mrs. Pillow, had achieved. Every penny had been given willingly, and in many cases more was given than was asked. No patient had been turned away who could not give. They had secured the College buildings (offered to them some years ago at £18,000) for only £6,750. The money had been advanced by their bankers, and they hoped to pay off this loan with the assistance of those who realised the value of medical education and research.

Referring to the decision of the hospital to stand out of the Combined Appeal, Mr. Verity said that they disagreed with this method of raising funds, by bazaars, processions and entertainments. Nearly always the results were not commensurate with the expenses incurred. At the best an aftermath of slackness was left among the philanthropic public. In their opinion, the Combined Appeal was being made at the wrong time. Trade could not be worse, taxes could not be higher, and to screw an extra £500,000 out of the benefactors of the London hospitals, or, rather, try to, at such a time was useless. Again, the Combined Appeal was intended largely to pay off the deficits of the London hospitals. They had no deficit there, and, without immodesty, they thought it was due to careful finance. Therefore, where would they come in? The Combined Appeal had cut right across the neighbourhood which so loyally supported that hospital in these hard times, and already they felt the decrease which was bound to occur to their yearly income.

### THE COMBINED APPEAL v. MR. VERITY.

IT would be a mistake to make too much of the decision of Charing Cross Hospital to stand outside the Combined Hospitals Appeal. Mr. Verity, the hospital's chairman, and his committee have managed their own affairs with great success, but, without minimising their efforts or reflecting on their policy, it is also true that a hundred and one circumstances affect the position of each hospital, and that, strictly speaking, no hospital is entitled to all the praise or all the blame for the situation in which it finds itself. Charing Cross Hospital, indeed, has had an uphill fight in recent years to gain its present prosperity, which is therefore all the more creditable; and since the majority of the London hospitals have decided to pool their resources in the present appeal, the abstention of one should not affect the result. On general grounds, we feel bound to support every step towards hospital co-ordination in London, and the present appeal is, strictly speaking, an experiment to test the value of combination in matters of appeals and finance. If it succeeds unmistakably, the way may be opened to closer combination in other directions, where it is certainly needed. Had the decision of one hospital to abstain affected the chances of general success, we should have become stern critics of the abstainer. But the course which it has chosen can affect itself only, and its neighbours would have been weakened by the presence of an unwilling co-operator.

### SUBSCRIPTIONS AND CITY FIRMS: A PRACTICAL PROPOSAL.

SIR GEORGE LAWSON JOHNSTON, in his speech at the annual meeting of Estates Control, Ltd., drew attention to the pressing question of rates in London, and reminded his audience of business men of the further burden that would fall upon them if the voluntary hospital system were imperilled. He went on to propose that the question of a hospital subscription should be put on the agenda for consideration at the next board meeting of every company or firm that pays rates or taxes in London. Mr. Peto Bennett said that from a financial point of view it would certainly be in the interests of the great companies of London, and if their contribution took the form, for instance, of a percentage of profits, a very small figure in each case would suffice. Synchronising with the Hospitals Combined Appeal, the proposal is doubly valuable, and we hope that it may be generally considered and eventually adopted. Sir George Lawson Johnson has done excellent service in bringing it forward at an annual meeting of shareholders. Our experience is that once the difficulty of finding a man of business who will make such suggestions is overcome, the problem of carrying them out is already half accomplished. Many attempts have been made to interest city firms in the voluntary hospitals, but no more comprehensive effort has been proposed than to place a hospital subscription on the agenda of every company meeting. The moment should not be lost, now that the hospital appeal is in the minds of every Londoner.

### ANNUAL SUBSCRIPTIONS IN KIND.

WHILE outstanding accounts are in themselves a nuisance to both parties concerned in them, they become doubly so when the hospital that owes the money is also living on an overdraft. At King's College Hospital, for example, it is understood that cheques can be issued only six months after they have been drawn. This has led one firm to cancel the bill, and to ask the hospital to regard the sum involved as a donation. We can well believe that this solution is grateful to both parties, and that the forbearance shown by the firm is even a convenience to itself. Indeed, the general question of hospital overdrafts and outstanding accounts leads to a further suggestion, whether it might not be possible to organise annual subscriptions in kind from those firms which provide a hospital with its necessities. Gifts in kind are always available from private donors, especially perishable goods, and particularly in the provinces; for hospital pound days tradesmen and others have often given manufactured articles. Why then not consider the possibility of annual subscriptions in kind from firms and manufacturers? The details would require thinking out, but probably it would cost the subscribing firms less than a subscription in money and benefit the hospital, perhaps, more. There are too many things in its favour to make initial difficulties prohibitive. The practical point is to show cause why it should not be done.

---

The bowlers of North Staffordshire during the past season made a special effort in aid of the funds of the Orthopædic Hospital, Stoke-on-Trent, raising the sum of £848 15s. Mr. Victor Johnson, Secretary of the North Staffordshire Cripples' Aid Society, who acted as hon. secretary to the effort, is now endeavouring to get other sports to follow suit. The bowlers hope to make a further effort this season.

An anonymous donor lately sent £5 to the Royal National Orthopædic Hospital for benefits received there in 1840.

The fund for the Woolwich War Memorial Hospital has reached £89,000.

A fête in aid of the Princess Alice Memorial Hospital, Eastbourne, was lately opened by the Duke of Devonshire. It is hoped to raise £2,000.

The new convalescent home at Grindon, Sunderland, has been formally opened for women and children selected by the Guild of Help. Colonel Maurice Moore raised a fund, which has enabled the house to be rented and suitably furnished.

A sum of £1,400 has already been subscribed toward the new Nurses' Home at Arbroath Infirmary, for which £5,000 is required. A conditional gift of £200 is. promised if the new building is opened free of debt.

A sum of £750 was raised from the ten days' public inspection of the Mystery Tower at Shoreham, for the benefit of the Royal Sussex County Hospital. Major Sexton bore the whole of the expenses, which amounted to some £500.

The Birmingham Hospital Saturday Fund expects to have raised £53,C00, which, at a time of trade depression, compares well with the £60,338 collected last year. A revival of trade, it is thought, would increase the total to the £100,CC0 hoped for.

The contribution scheme connected with the Norfolk and Norwich Hospital has so developed that whereas two years ago it began with 70 branches and an income of £1,000, to-day there are 7C0 parish and factory funds and an income of £13,000.

Through the estate of the late Mrs. Hannah Tiffany, Leeds General Infirmary will receive a sum of £20,CC0. The legacy came as a very pleasant surprise, for the deficit at the end of last year was £35,0C0, and the present excess of expenditure averages £70 a day.

By the end of June grants to the value of £214,528 had been approved from the sum set apart by the Government in aid of the voluntary hospitals. Sir Alfred Mond has asked provincial hospitals to present their claims promptly, a hint of which the Local Committees will doubtless take note.

The Millpond at Southend Village, re-christened Peter Pan's Pool, is to be the scene of various water sports, and the adjacent grounds are to be laid out. A percentage of the receipts will benefit Great Ormond Street Hospital for Children, on a plan devised by Major Pat A'Beckett.

So generally has the hospital contribution scheme been adopted in the city and district, that most of the patients in Yarmouth Hospital are exempt from maintenance charges, though £544 was received from this source last year. The contribution scheme has raised nearly £2,000 in the same period.

The decision to build a new nurses' home at St. Marys' Hospital for Women and Children, Plaistow, is a fitting reward for the efforts to raise the necessary funds that have occupied Miss Kate Ray, the matron, for several years. The present quarters are described as "horse-boxes." A tender for £24,6C0 has been accepted.

The Nurses' Bazaar to provide further accommodation for the staff of the Liverpool Royal Infirmary raised £1,000 on the first day. Six humorous postcards, drawn by Mr. Mosscrop, the caricaturist, are to be permanently on sale for the benefit of the hospital. A wireless telephone was one of the popular attractions of the Bazaar.

The reopening of the Richard Murray Hospital at Benfieldside, which was founded in 1911 by the generosity of the donor whose name it bears, marks its return from the War Office to civil use. By arrangement with the County Council, the hospital will devote the top floor to maternity and child-welfare. Mr. John George Murray, in declaring the hospital re-opened, said the X-ray-room was at present without an installation, and he would have pleasure in providing one. The county medical officer, Dr. Hill, declared at the opening that not even the Royal Victoria Infirmary at Newcastle was better equipped.

